# Feasibility of intraoperative ultrasound of the small bowel during Crohn’s disease surgery

**DOI:** 10.1007/s10151-020-02268-9

**Published:** 2020-06-23

**Authors:** V. Celentano, R. Beable, C. Ball, K. G. Flashman, R. Reeve, C. Fogg, M. Harper, A. Higginson

**Affiliations:** 1 Queen Alexandra Hospital, Portsmouth Hospitals NHS Trust, Portsmouth, UK; 2 University of Portsmouth, Portsmouth, UK; 3 University of Southampton, Southampton, UK

**Keywords:** Crohn’s disease, Intraoperative ultrasound, Inflammatory bowel disease, Colorectal surgery

## Abstract

**Background:**

Intraoperative assessment of the extent and location of Crohn’s disease is not standardised and relies on a mixture of surgeons’ experience, tactile feedback and macroscopic appearance. To overcome this variability, we developed a protocol for full intraoperative ultrasound scan of the small bowel and we here report the results of “Assessing the Feasibility and Safety of Using Intraoperative Ultrasound in Ileocolic Crohn’s Disease—The IUSS CROHN Study”.

**Methods:**

This is a prospective single centre observational study with enrolment of all patients undergoing elective surgery for terminal ileal Crohn’s disease from January 2019 to March 2020. Patients underwent laparoscopic ileocolic resection, according to a standardised technique. Ultrasound intraoperative quantitative assessment was performed according to the METRIC (MREnterography or ulTRasound in Crohn’s disease) scoring guide.

**Results:**

Intraoperative ultrasound was successfully performed in 6 patients from the ileocaecal valve to the proximal jejunum. The median time required was 23.5 min (range 17–37 min) as compared to 6.5 min (5–12 min) required for the macroscopic evaluation performed by the surgeon. In 3 patients, intraoperative ultrasound identified more disease than surgical evaluation.

**Conclusions:**

This feasibility study demonstrated the safety of intraoperative ultrasound and allowed the development of a standardised protocol for intraoperative ultrasound and the data collection required to inform a randomised multicentre study.

## Introduction

Multiple imaging modalities are often required to assess severity and extent of Crohn’s disease (CD), considering its multifocal and relapsing nature, with magnetic resonance imaging (MRI) representing the preferred technique to aid surgical decision making in view of the high sensitivity for detection of proximal lesions and preoperative mapping [1].

On the other hand, intraoperative assessment of the extent and location of CD is not standardised and relies on a mixture of surgeons’ experience, tactile feedback, macroscopic appearance and preoperative imaging. This can result in interobserver variability, affecting the length of small bowel removed at surgery and the management of occult disease. Moreover, incorrect intraoperative mapping of the extent of disease may mislead the multidisciplinary team in decision making about postoperative maintenance treatment. To overcome this variability, we developed a protocol for full intraoperative ultrasound scan (USS) of the small bowel [2]. The aim of this study was to report the results of the feasibility study “Assessing the Feasibility and Safety of Using Intraoperative Ultrasound in Ileocolic Crohn's Disease—The IUSS CROHN Study”.

## Materials and methods

### Study settings and eligibility criteria

Assessing the Feasibility and Safety of Using Intraoperative Ultrasound in Ileocolic Crohn's Disease (The IUSS CROHN Study—NCT03939117) is a prospective single centre observational study with enrolment of patients from January 2019 to March 2020.

We included patients with CD affecting the small bowel requiring elective surgical treatment, according to the following inclusion criteria: (1) Aged 18 years or over; (2) American Society of Anaesthesiologists (ASA) grade I, II or III; (3) Undergoing elective surgery to remove part of the terminal ileum which is affected by CD; (4) Indication for surgery agreed at inflammatory bowel disease (IBD) multidisciplinary team (MDT) meeting; (5) Able to give written informed consent.

Patients were excluded in case of pregnancy, emergency surgery or history of previous abdominal surgery for CD.

### Intraoperative ultrasound protocol

**Fig. 1:**
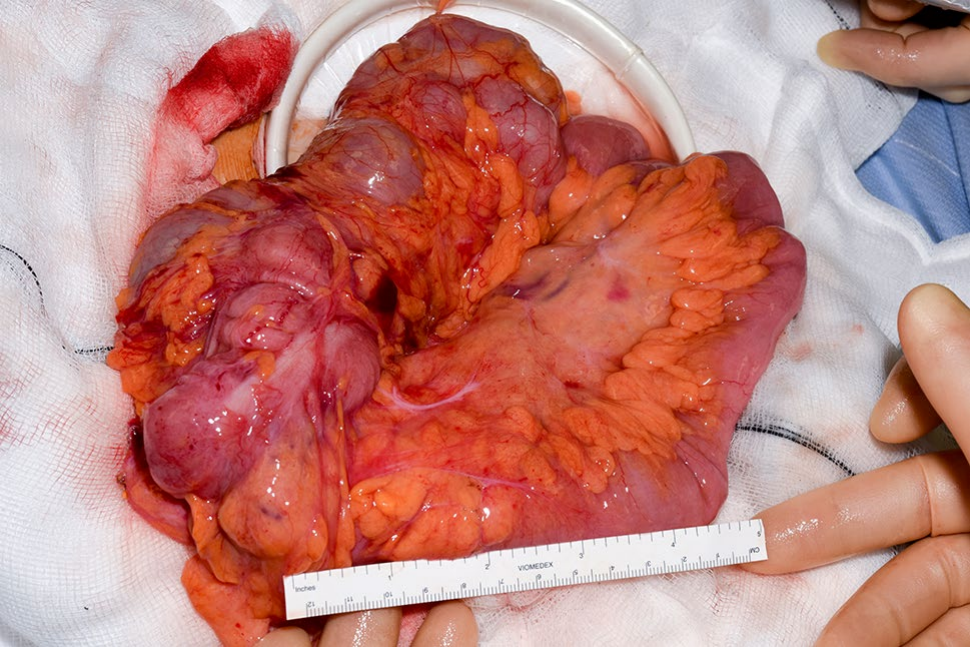
Macroscopic evaluation of the small bowel performed by the surgeon

Patients underwent laparoscopic ileocolic resection, according to a standardised technique, with full mobilisation of the hepatic flexure following a medial to lateral approach. The specimen was delivered via a service midline laparotomy through a wound protector, for extracorporeal division of the mesentery and anastomosis. Where present, internal fistulae were divided intracorporeally. Initially, as per gold standard procedure, the full length of the small bowel from duodenojejunal flexure to ileocaecal valve was assessed macroscopically by the operating surgeon (Fig. 1) and the areas of CD to be resected identified with a sterile surgical marking pen on the antimesenteric border of the small bowel. The presence and location of other sites of disease was documented.

**Fig. 2:**
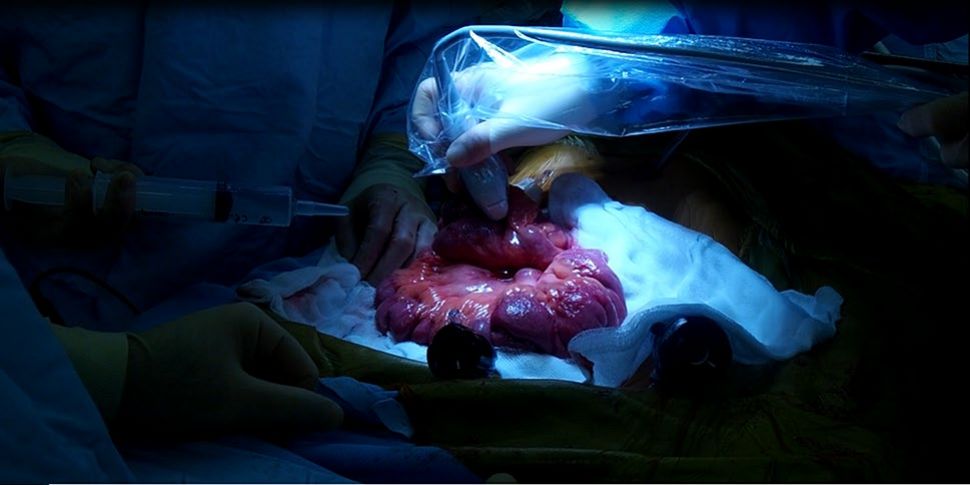
Intraoperative ultrasound scan

**Fig. 3:**
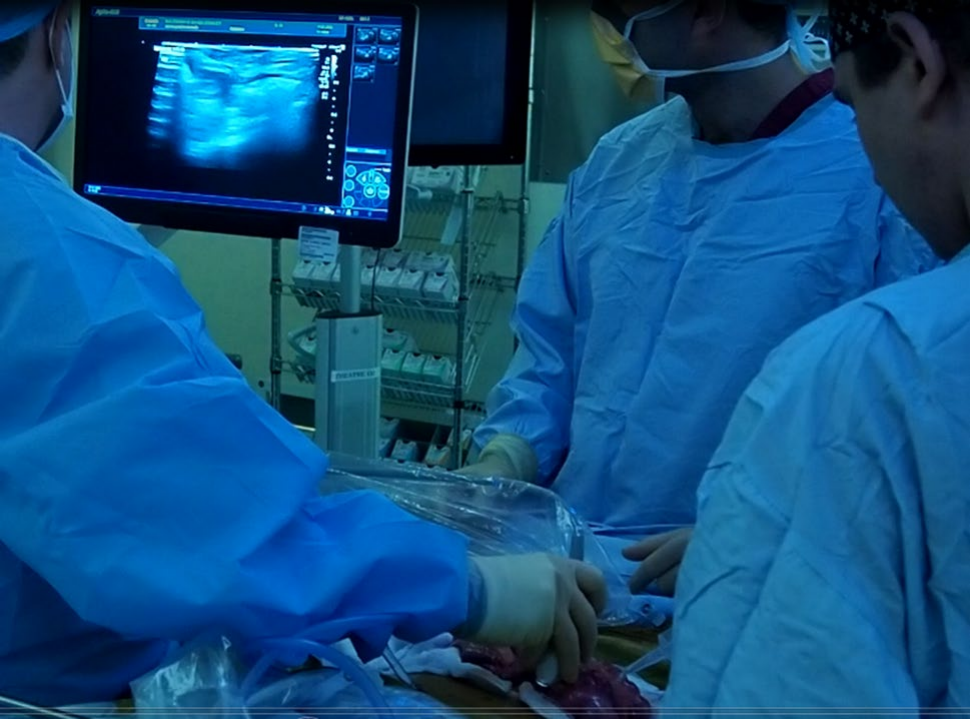
Intraoperative ultrasound scan. Set up

**Fig. 4:**
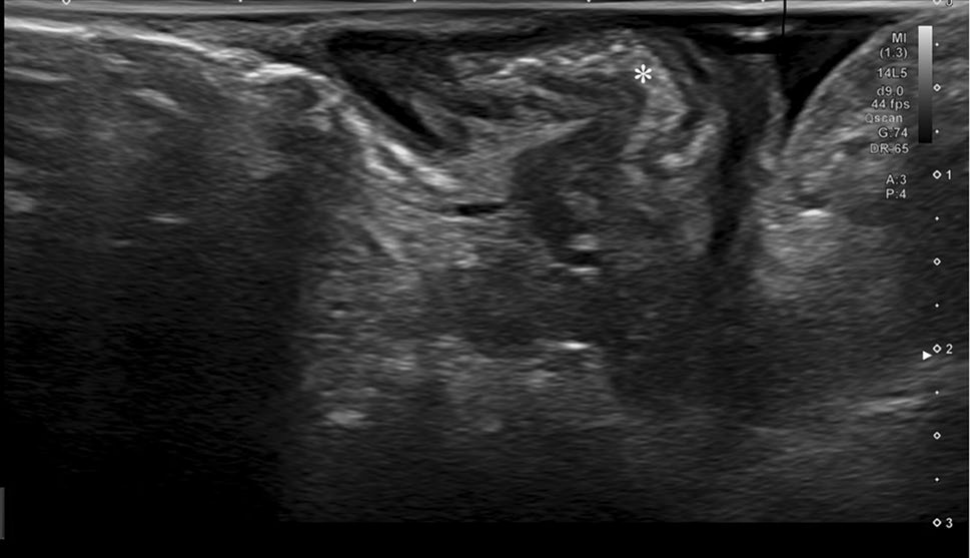
Skip lesion detected on intraoperative ultrasound. *Thickening of the mucosa and submucosa of the small bowel

**Fig. 5:**
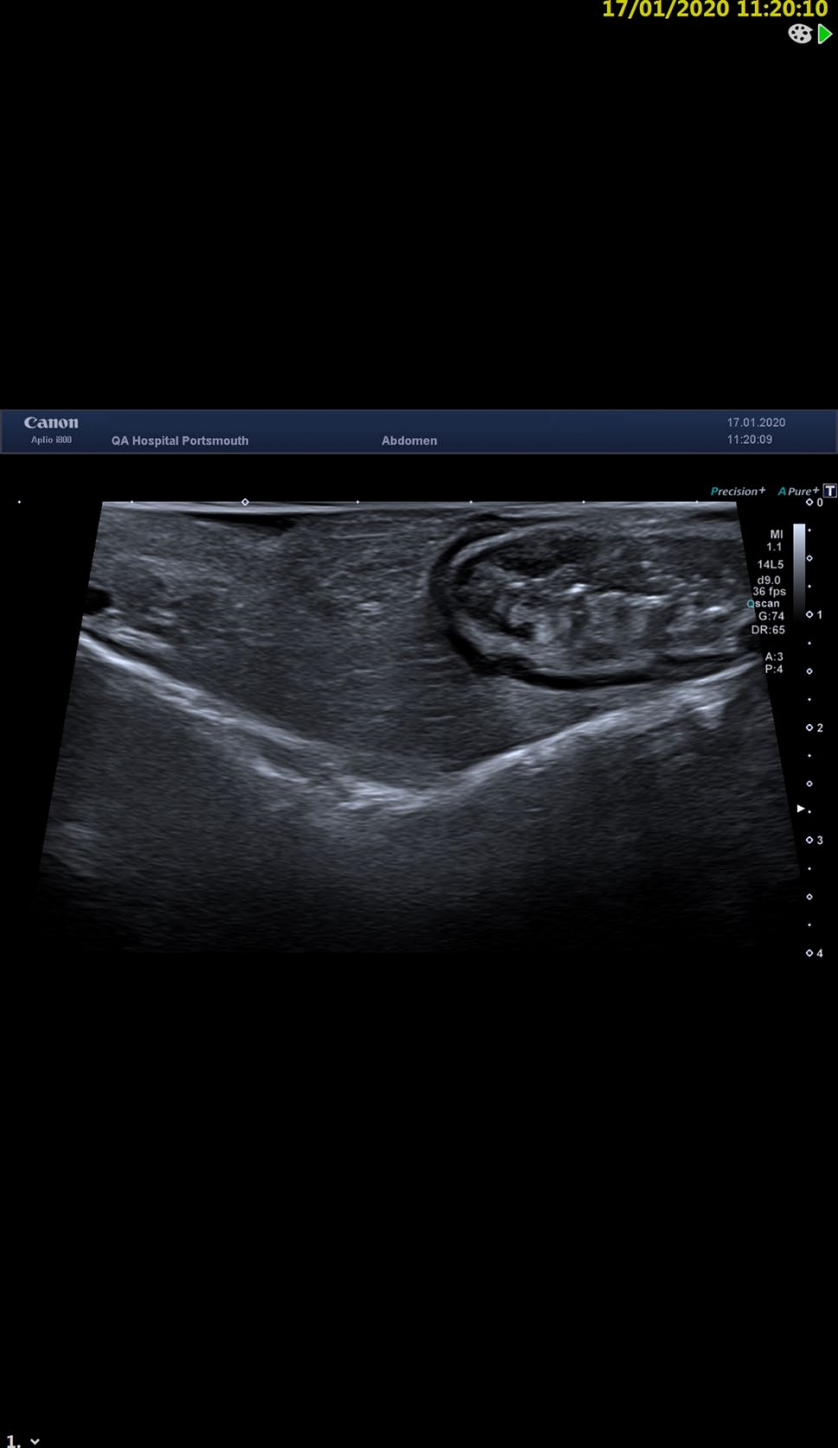
Mesenteric thickening on intraoperative ultrasound. *Thickening of the small bowel mesentery

The intervention assessed in the study was the use of intraoperative USS of the small bowel. A gastrointestinal consultant radiologist, with expertise in CD imaging and abdominal USS repeated the full intraoperative assessment of the small bowel, by applying directly on the bowel a sterile USS probe, prior to resection being performed by the surgeon [3]. Acoustic coupling was obtained by irrigating the bowel with sterile saline (Figs. 2, 3). The radiologist marked the extent of disease on the mesenteric border of the small bowel, blinded to the findings documented by the surgeon on the opposite side of the bowel. The USS quantitative assessment was performed according to the METRIC (MREnterography or ulTRasound in Crohn’s disease) scoring guide [4]: presence and number of lymph nodes, bowel wall thickness, functional obstruction, submucosal layer thickness (Fig. 4), echogenicity and clarity, mucosal layer thickness, mesenteric fat echogenicity (Fig. 5), ulceration, Doppler vascular pattern and peristalsis related to stricturing.

The findings of the surgeon and the radiologist were documented, focusing on the length of small bowel affected by disease in centimetres, and location and extent of other sites of disease, with reference distance estimated in centimetres from the landmarks of the duodenojejunal flexure and ileocecal valve. The extent of resection was decided intraoperatively by the surgeon according to the “gold standard” of care, which currently is macroscopic evaluation and tactile feedback performed by the surgeon. No changes occurred in this feasibility study to the length of bowel which was resected, as to date, macroscopic evaluation by the surgeon is the worldwide accepted standard practice. However, in order not to miss any significant findings, before performing the resection the surgeon was always made aware of the findings of the radiologist and reviewed the small bowel to make sure the most appropriate option was chosen for the patient in case of discrepancies.

### Study objectives and endpoints

The study objective was to evaluate the feasibility of using intraoperative USS in patients with CD of the small bowel, to describe the steps of the procedure and to assess the safety of the USS procedure during surgery.

Endpoints of this qualitative study were:

- Number of intraoperative USS assessments successfully completed;
- Number of operations in which key information was provided by the USS that altered the surgical intervention;
- Adverse events during USS;
- Operating time, intra- and postoperative complication rates, 30-day morbidity and mortality rate;
- Quantitative assessment of additional operating time required in theatre to perform the intraoperative USS.

### Statistical analysis

Categorical variables are presented as frequency or percentage and were compared with the use of the chi-square test or Fisher’s exact test, as appropriate. Continuous variables are presented as means (± standard deviation) and were compared with the use of Student’s *t* test. The Mann–Whitney *U* test was used for continuous, not normally distributed, outcomes.

### Ethics

The study protocol was reviewed and approved by the Wessex Ethics Committee. Informed consent was obtained from the patients.

## Results

Six USS were performed with no intraoperative complications. The scan was successfully performed in all patients from ileocaecal valve to proximal jejunum (Table 1) via a 4 cm periumbilical incision (range 3–7). In one patient, it was necessary to resect the bulky ileocaecal specimen before the USS of the proximal bowel could be safely completed.

**Table 1:** Patients’ baseline data and surgical outcomes

Male:female ratio	4:2
Age (years)	31 (25–61)
Smoking	1 (16.7%)
Medical treatment	AZA: 2 (33.3%)
	Steroids: 2 (33.3%)
Preoperative blood test	Alb (g/L): 38 (35–41)
	Hb (g/L): 131 (94–157)
	CRP (mg/L): 8.5 (3–13)
	WCC (10^9^/L): 8 (5.8–9.7)
Bradshaw index	Preoperative: 8 (4–10)
	Postoperative: 2 (0–8)
SIBDQ	Preoperative: 35.5 (24–57)
	Postoperative: 60 (34–69)
Disease pattern	Stricturing: 3
	Penetrating: 2
	Non stricturing non penetrating: 1^a^
Perianal disease	2 (33.3%)
Ileostomy	None
Conversion	None
Operating time (min)	184 (174–225)
Morbidity	1 (16.7%)
LOS (days)	5 (4–8)

The data are expressed as median (range) or number (percentage)

*AZA *azathioprine, *Alb *albumin, *CRP *C-reactive protein, *WCC *white cell count, *SIBDQ *short inflammatory bowel disease questionnaire, *LOS *postoperative length of hospital stay

^a^Surgery indicated for lack of response to maximum medical treatment

The intraoperative USS required a median time of 23.5 min (range 17–37 min) as compared to 6.5 min (5–12 min) required for the macroscopic evaluation performed by the surgeon.

The intraoperative USS identified more disease than the surgical evaluation in 3 patients: in 2 patients more skip lesions were identified (2 in 1 patient and 1 in another patient and these required no change in the surgical strategy as there was no obstruction) and in 1 patient a critical stricture at the ileocaecal valve was identified and treated rather than preserved as per macroscopic evaluation.

When compared with the preoperative MRI scan, the intraoperative USS identified 2 additional skip lesions in 1 patient and 1 additional skip lesion in another patient.

All 6 patients underwent laparoscopic ileocaecal resection with no conversions to open surgery. In 3 patients, Heineke–Mikulitz strictureplasties were also required for the treatment of proximal disease (3 in 2 patients and 1 in another patient). In 1 patient, an additional small bowel resection was needed, whilst another patient required a suture repair of an ileosigmoid fistula. Only 1 patient developed postoperative complications, which were a wound and chest infection. There were no readmissions or reoperations within 30 days of surgery.

## Discussion

Intraoperative USS of the entire small bowel was safely and successfully performed in 6 patients, enhancing the macroscopic assessment performed by the surgeon by providing additional information in 2 out of 6 cases and altering the surgical strategy in 1 patient. The intraoperative scan added approximately 15 min to the gold standard macroscopic evaluation performed by the surgeon, but the expectation is that the extra time required might be reduced over time as the team performs more cases and progresses through the learning curve.

This feasibility study demonstrated the safety of intraoperative USS and has allowed the development of a standardised protocol for intraoperative USS, also obtaining the required data to inform a multicentre study. It is unlikely that intraoperative USS will affect the decision making in the majority of the patients, nevertheless, double checking the bowel may not only further enhance IBD surgeons’ skills, by providing real time direct feedback on the presence and severity of disease, but also improve the evaluation of the small bowel by detecting more occult disease, which might have been missed by the preoperative imaging and macroscopic assessment, resulting in better informed multidisciplinary decision making on postoperative recurrence and maintenance treatment. Even if it was not one of the main study objectives, intraoperative USS compared favourably even with preoperative MRI enterography. Moreover, with intraoperative USS it was possible to evaluate the proximal small bowel too and not only on the distal ileum.

We advocate a standardised approach to intraoperative evaluation of extent and location of CD, based on reliable and reproducible techniques, minimising the risk of surgical recurrence, optimising decision making on maintenance treatment and follow-up and protecting patients from unnecessary extended small bowel resections. We are planning a multicentre study to further evaluate the role of intraoperative USS during CD surgery and long-term follow-up.
