# Correction to: Feasibility of intraoperative ultrasound of the small bowel during Crohn’s disease surgery

**DOI:** 10.1007/s10151-023-02760-y

**Published:** 2023-02-06

**Authors:** V. Celentano, R. Beable, C. Ball, K. G. Flashman, R. Reeve, C. Fogg, M. Harper, A. Higginson

**Affiliations:** 1 Queen Alexandra Hospital, Portsmouth Hospitals NHS Trust, Portsmouth, UK; 2 University of Portsmouth, Portsmouth, UK; 3 University of Southampton, Southampton, UK

**Correction to: Techniques in Coloproctology (2020) 24:965–969**

 10.1007/s10151-020-02268-9

In this article the wrong figure appeared as Fig. 5; the figure should have appeared as shown below.

**Fig. 5:**
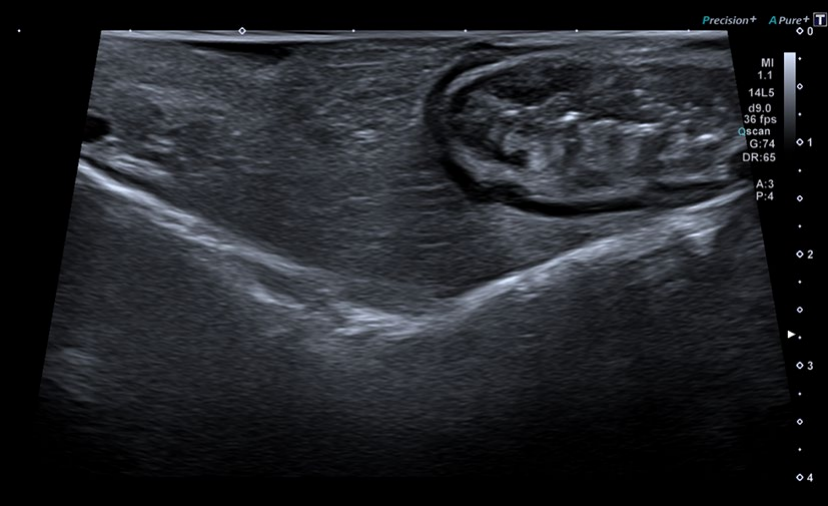
Mesenteric thickening on intraoperative ultrasound. *Thickening of the small bowel mesentery

The original article has been corrected.

